# Rapamycin enhances CAR-T control of HIV replication and reservoir elimination in vivo

**DOI:** 10.1172/JCI185489

**Published:** 2025-02-11

**Authors:** Wenli Mu, Shallu Tomer, Jeffrey Harding, Nandita Kedia, Valerie Rezek, Ethan Cook, Vaibahavi Patankar, Mayra A. Carrillo, Heather Martin, Hwee Ng, Li Wang, Matthew D. Marsden, Scott G. Kitchen, Anjie Zhen

**Affiliations:** 1 Division of Hematology/Oncology, Department of Medicine, and; 2 UCLA AIDS Institute and the Eli and Edythe Broad Center of Regenerative Medicine and Stem Cell Research, David Geffen School of Medicine at UCLA, Los Angeles, California, USA.; 3 Department of Microbiology & Molecular Genetics and; 4 Division of Infectious Diseases, Department of Medicine, School of Medicine, University of California, Irvine, Irvine, California, USA.

**Keywords:** AIDS/HIV, Immunology, Immunotherapy, T cells

## Abstract

Chimeric antigen receptor (CAR) T cell therapy shows promise for various diseases. Our studies in humanized mice and nonhuman primates demonstrate that hematopoietic stem cells (HSCs) modified with anti-HIV CAR achieve lifelong engraftment, providing functional antiviral CAR-T cells that reduce viral rebound after antiretroviral therapy (ART) withdrawal. However, T cell exhaustion due to chronic immune activation remains a key obstacle to sustained CAR-T efficacy, necessitating additional measures to achieve functional cure. We recently showed that low-dose rapamycin treatment reduced inflammation and improved anti-HIV T cell function in HIV-infected humanized mice. Here, we report that rapamycin improved CAR-T cell function both in vitro and in vivo. In vitro treatment with rapamycin enhanced CAR-T cell mitochondrial respiration and cytotoxicity. In vivo treatment with low-dose rapamycin in HIV-infected, CAR-HSC mice decreased chronic inflammation, prevented exhaustion of CAR-T cells, and improved CAR-T control of viral replication. RNA-sequencing analysis of CAR-T cells from humanized mice showed that rapamycin downregulated multiple checkpoint inhibitors and upregulated key survival genes. Mice treated with CAR-HSCs and rapamycin had delayed viral rebound after ART and reduced HIV reservoir compared with those treated with CAR-HSCs alone. These findings suggest that HSC-based anti-HIV CAR-T cells combined with rapamycin treatment are a promising approach for treating persistent inflammation and improving immune control of HIV replication.

## Abstract 2

Rapamycin enhances CAR-T cell function, reduces HIV-mediated chronic inflammation and T cell exhaustion, delays viral rebound and reduces viral reservior, offering a promising strategy for improving immune control of HIV replication in vivo.

## Graphical abstract

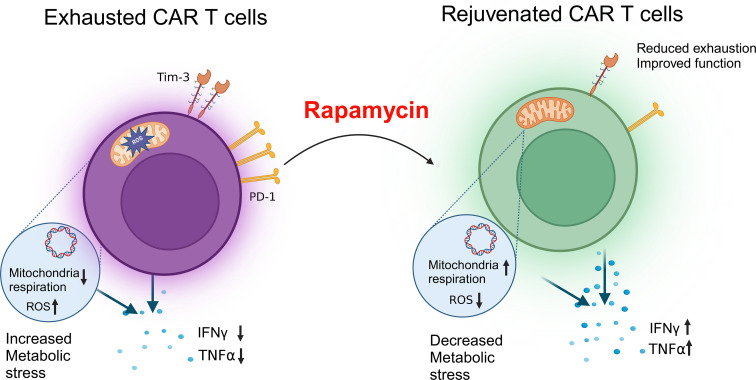

## Introduction

Engineering T cells with anti-HIV chimeric antigen receptors has emerged as a promising gene therapy strategy to control HIV infection. HIV-specific CD8^+^ cytotoxic T lymphocytes (CTLs) are essential in suppressing HIV replication and eliminating HIV-infected cells (1, 2). However, because of immune evasion by HIV (3) and development of dysfunctional HIV-specific T cells, natural CTLs are incapable of complete control of HIV replication in the absence of combination antiretroviral therapy (ART) (4) and cannot eliminate reservoirs with “kick-and-kill” HIV cure strategies (5). A promising approach to overcome these barriers is through chimeric antigen receptor (CAR) engineered T cell therapy (6). T cells engineered with CD4-based CARs, which use CD4 extracellular domains to recognize HIV-1 envelope (Env), can effectively kill HIV-infected cells and limit viral escape, as an escape from CD4 recognition would directly decrease viral fitness (7, 8). However, persistence, trafficking, and maintenance of function remain major challenges for peripheral CAR-T cell therapy (9). To overcome these issues, we showed that CAR-modified hematopoietic stem cells (HSCs) are capable of lifelong engraftment and allow development of functional CAR-T cells in vivo (10–12). Our studies in humanized mice (10, 11) and nonhuman primates (NHPs) (12, 13) demonstrated the feasibility and efficacy of the CAR-HSC therapy and showed that CAR-HSC–transplanted animals have reduced viral rebound after ART withdrawal. We have also made substantial improvements to this therapy by modifying the original CD4CAR construct to a second-generation CAR, termed D1D2CAR41BB. We showed that D1D2CAR41BB-HSC–transplanted animals have improved CAR-T cell differentiation, better CAR-T cell persistence, and enhanced viral control (10). However, our lead CAR-HSC therapy still fell short of achieving complete viral remission in the absence of ART.

T cell exhaustion remains a major challenge for CAR-T cell therapy for HIV-1 cure. Driven by chronic immune activation, T cell exhaustion remains one major barrier to achieving sustained immune surveillance for viral infection and cancer (14). Despite our recent successes in HSC-based CAR therapy, we found that HSC-derived D1D2CAR41BB T cells are also subject to becoming exhausted. Similar CAR-T cell exhaustion was also observed during peripheral anti-HIV CD4CAR-T cell therapy in NHP models (15, 16). Although antibodies targeting immune checkpoint blockades (ICBs) (such as PD-1 blockade) may restore CAR-T cell function transiently (16, 17), ICB treatment can lead to serious side effects, such as onset of type 1 diabetes and colitis and other adverse effects (18, 19) that may be unacceptable to that may be unacceptable to ART-treated people living with HIV (PLWH). Therefore, alternative strategies to safely reverse or prevent CAR-T cell exhaustion are critical for achieving long-term control of HIV replication.

Rapamycin, which inhibits mammalian target of rapamycin (mTOR) complex 1, was first approved for use in anticancer therapies and transplant rejection prevention. In recent years, rapamycin has shown robust geroprotective ability, extending lifespans in multiple model organisms, including the lifespan of genetically heterogeneous mice from multiple research groups (reviewed in ref. 20). In humans, rapamycin administration has been shown to reverse immunosenescence and boost response to seasonal flu vaccines (21, 22), and it is being studied in multiple clinical trials in healthy individuals and individuals with age-related diseases (23–25). Importantly, rapamycin can lead to metabolic reprogramming in T cells, shifting metabolism from glycolysis to oxidative phosphorylation and modulating lipid metabolism to enhance CD8^+^ T cell memory formation (26, 27). While chronic treatment of humans with high doses of rapamycin or analogs is associated with deleterious metabolic effects, recent studies in humans have shown that a lower or intermittent dosing regimen of mTOR inhibitors in older adults is well tolerated and leads to improved immune function and reduced infection in the elderly (21, 22, 28). A recent study also demonstrated that rapamycin treatment during the beginning of chronic infection improves CD8^+^ T cell memory formation and the efficacy of PD-1–targeted therapy (29). Moreover, our recent work showed that intermittent in vivo treatment of HIV^+^ humanized mice with rapamycin led to reductions in immune activation and improved endogenous anti-HIV T cell function, resulting in accelerated viral suppression during ART and reduced viral rebound after ART withdrawal (30). Here we show that low-dose, intermittent rapamycin restores and improves anti-HIV CAR-T cell function during chronic HIV infection. We found that rapamycin treatment notably remodeled the CAR-T cell transcriptome and improved mitochondria function, resulting in enhanced antiviral activities of CAR-T cells. This led to delayed viral rebound after ART withdrawal and improved viral control by CAR-T cells, suggesting potential therapeutic value of rapamycin in improving CAR-T cell function in vivo.

## Results

### Rapamycin treatment normalizes anti-HIV CAR-T mitochondria metabolism and improves CAR-T function in vitro.

**Figure 1:**
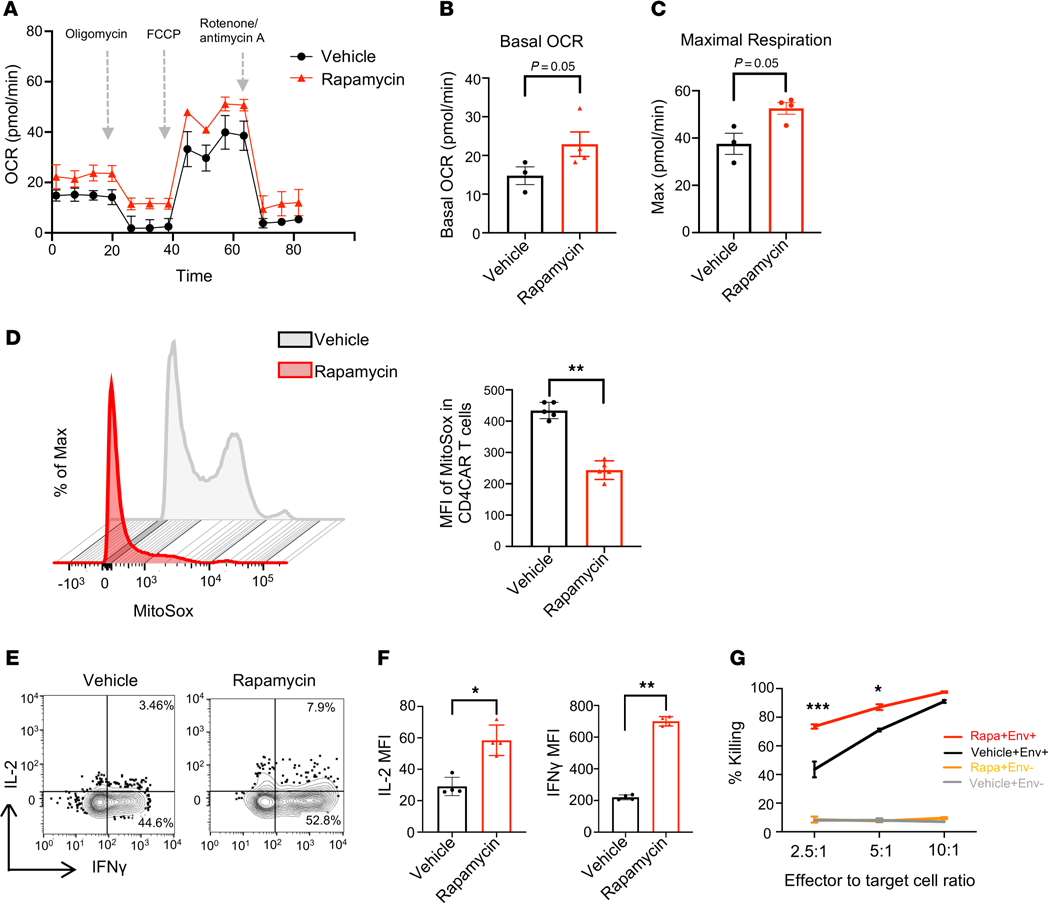
Treatment of anti-HIV CAR-T cells with rapamycin modifies cellular metabolism in vitro. Anti-HIV CD4CAR-T cells were produced by transduction of activated primary PBMCs from healthy donors. Cells were then sorted to greater than 90% CAR^+^ purity and expanded using 100 IU/mL IL-2 for 2 weeks to promote exhaustion, followed by treatment with either DMSO or 50 pM of rapamycin for 2 days. Afterward, Seahorse assay was performed on treated CAR-T cells. (**A**) The oxygen consumption rate (OCR) over time changes under basal metabolic conditions and in response to metabolic inhibitors. (**B**) Basal OCR levels. (**C**) Maximal respiratory levels. (**D**) ROS were analyzed in CD4CAR-T cells labeled with MitoSOX after treatment as shown by flow cytometry and MFI summary of MitoSOX. (**E** and **F**) Cytokine assay. Vehicle- or rapamycin-treated CAR-T cells were cocultured with HIV Env–expressing (stimulated ACH2) cells overnight, followed by GolgiPlug (BD Biosciences) for 6 hours. Percentages of IFN-γ and IL-2 expression were measured by flow cytometry in CD4CAR-T cells. Representative flow plot and summary are shown. (**G**) Killing assay. Vehicle- or rapamycin-treated CAR-T cells were cocultured with either HIV Env^+^ (stimulated ACH2) or HIV Env^–^ (unstimulated ACH2) cells overnight at 2.5:1, 5:1, and 10:1 ratio. Specific killing activity is shown for vehicle-treated and rapamycin-treated CAR-T cells. All values are means ± SD of at least 3 independent experiments. Mann-Whitney test (unpaired); *P < 0.05, **P < 0.01, ***P < 0.001.

To examine whether rapamycin modifies CAR-T cell metabolism and restores exhausted anti–HIV-1 T cell functions in vitro, we investigated the effects of rapamycin on D1D2CAR41BB-modified primary T cells that can recognize and kill HIV-infected cells as described previously (10–12). We generated CAR-T cells by activating primary PBMCs from HIV-seronegative individuals and transducing them with a CAR-expressing lentiviral vector. Afterward, CAR-T cells were cultured in vitro for 2 weeks with IL-2 to stimulate T cell proliferation and induce T cell exhaustion. After culture, cells were treated with either rapamycin or vehicle control for 2 days followed by mitochondrial respiration analysis using a Seahorse assay. As shown in Figure 1A, oxygen consumption rates (OCRs) over time were measured in the presence of metabolic inhibitors to assess the impact of rapamycin treatment on mitochondrial function in CAR-T cells. Basal OCR reflects oxygen consumption at rest (0–20 minutes), while maximal OCR measures capacity after uncoupling of the electron transport chain, allowing the mitochondria to operate at their maximal capacity without generating ATP (40–70 minutes). Rapamycin-treated CAR-T cells demonstrated improved basal (Figure 1B) and maximal mitochondrial respiration levels (Figure 1C), highlighting potential ability to rescue ATP-linked mitochondrial respiration in antiviral CAR-T cells. Rapamycin-treated CAR-T cells also showed a reduction in mitochondrial reactive oxygen species (ROS) by MitoSOX staining (Figure 1D). These results suggest that rapamycin can reduce ROS and enhance mitochondria functions in exhausted anti-HIV CAR-T cells. To investigate whether rapamycin has a restorative effect on anti–HIV-1 CAR-T cell function, we tested rapamycin-treated CAR-T cells’ ability to produce proinflammatory cytokines and cytotoxic capacity by coincubating CAR-T cells with control or target cells that express HIV Env. As shown in Figure 1, E–G, we observed significant increases in both IL-2 and IFN-γ production and improved cytotoxic killing activity by CAR-T cells treated with rapamycin. Taken together, these data strongly suggest that rapamycin treatment can remodel anti-HIV CAR-T cell metabolism and restore antiviral T cell function in vitro.

### HIV induces HIV-specific CAR-T cell exhaustion and dysfunction, while rapamycin treatment alleviates exhaustion and restores the viral suppression function of CAR-T cells.

**Figure 2:**
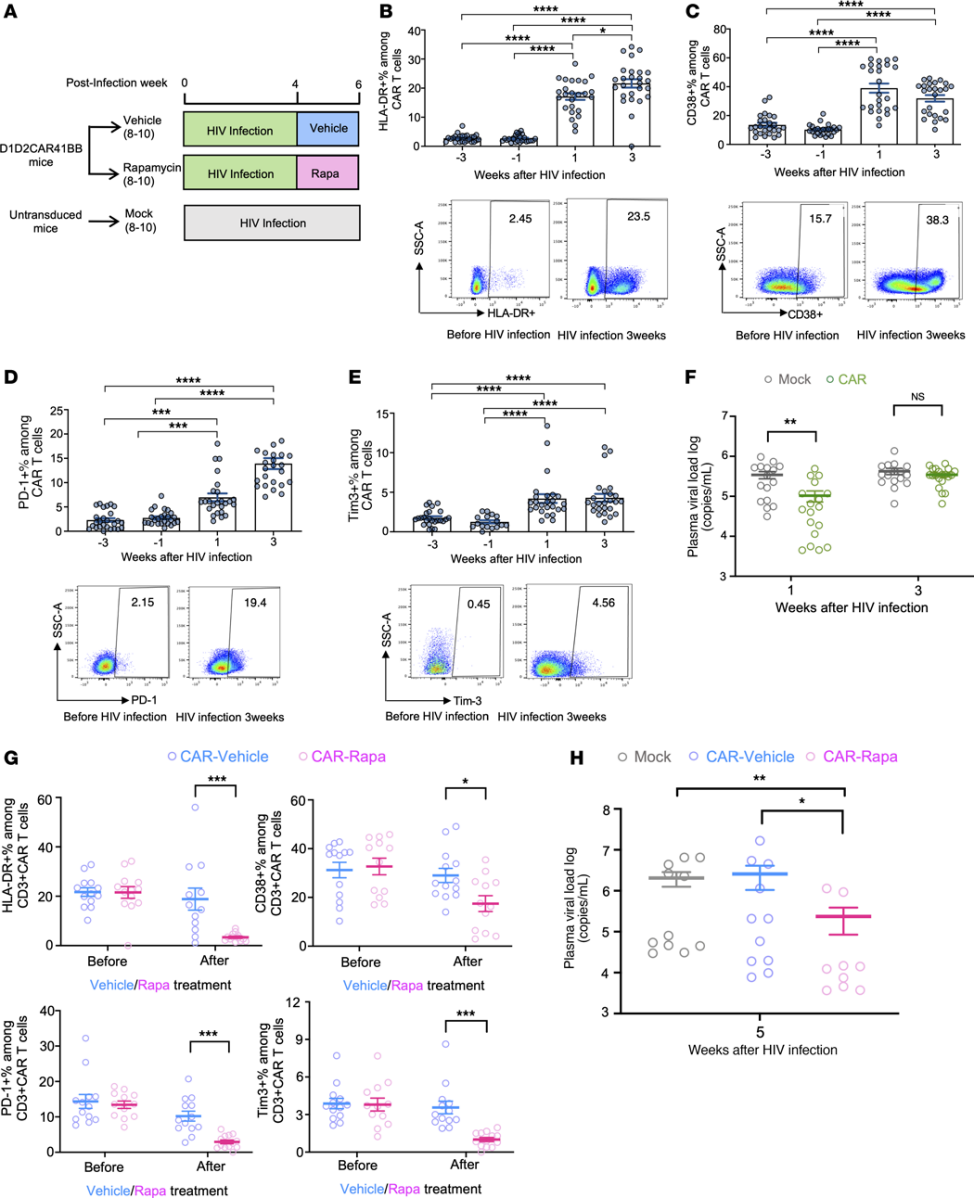
Chronic HIV infection leads to CAR-T cell exhaustion, while rapamycin treatment alleviates activation and exhaustion of CAR-T cells in vivo. (**A**) Humanized NSG-BLT mice were constructed with either unmodified HSCs or HSCs modified with D1D2CAR41BB. After immune reconstitution, mice were infected with HIV-1_NFNSXL9_. Four weeks after infection, mice with CAR-modified HSCs were treated with rapamycin or vehicle for 2 weeks. (**B**–**E**) Representative flow and average percentage of HLA-DR (**B**), CD38 (**C**), PD-1 (**D**), and Tim-3 (**E**) expression among CAR^+^CD3^+^ T cells before and after HIV infection as measured by flow cytometry (quantified by gating of percentage positive ± SEM) (*n* = 15–25 each group). (**F**) Plasma HIV viral load from mock or anti-HIV CAR mice at 1 week and 3 weeks of infection (*n* = 15–18 each group). (**G**) Average percentage of PD-1, Tim-3, HLA-DR, and CD38 expression among blood CAR^+^CD3^+^ T cells before and after rapamycin treatment (*n* = 10–15 each group). (**H**) Plasma HIV RNA copies from mock mice or CAR mice after 2 weeks of rapamycin or vehicle treatment (5 weeks after HIV infection) (*n* = 9–11 each group). The Mann-Whitney test was used to compare 2 groups, and the Kruskal-Wallis test was used for multiple comparisons (**B**–**E** and **H**). Each dot represents an individual mouse; horizontal bars indicate median values. *P < 0.05, **P < 0.01, ***P < 0.001, ****P < 0.0001.

Driven by chronic antigen stimulation and persistent immune activation, T cell exhaustion remains one major barrier to achieving sustained immune surveillance for chronic viral infection (31, 32). Similarly, CAR-T cell exhaustion is observed during peripheral anti-HIV CAR-T cell therapy in both humanized mice (15) and NHP models (16), undermining the efficacy of CAR-T cell therapy. Despite our recent successes in inhibiting HIV replication in humanized mice and NHPs using HSC-based CAR therapy (10–12) and improved CAR-T cell memory formation with a second-generation CAR containing the 41BB domain (10), viral loads rebounded in all CAR-HSC–transplanted animals after ART removal. Therefore, it is crucial to investigate immune exhaustion of HSC-derived CAR-T cells in chronic HIV infection in vivo. Hence, we constructed humanized bone marrow–liver–thymus (BLT) NSG mice with HSCs mock-transduced or transduced with lentiviral vectors expressing D1D2CAR41BB, as described previously (10). After immune reconstitution, we infected BLT mice with a high dose of HIV-1_NFNSXL9_ (500 ng of p24) to drive high viral loads and faster immune exhaustion (schematically shown in Figure 2A). As expected, we observed a significant and gradual increase in the activation markers HLA-DR (Figure 2B) and CD38 (Figure 2C) on CAR-T cells from peripheral blood after HIV infection (representative flow gating strategy is shown in Supplemental Figure 1; supplemental material available online with this article; https://doi.org/10.1172/JCI185489DS1). Three weeks after infection, we also observed a significant increase in the exhaustion markers PD-1 and Tim-3 among CAR-T cells (Figure 2, D and E). Consequently, while CAR mice demonstrated lower viral loads compared with mock 1 week after HIV infection, the viral suppression effects were lost by 3 weeks (Figure 2F), suggesting exhaustion/dysfunction of CAR-T cells.

We previously demonstrated that low-dose and intermittent rapamycin treatment decreases chronic inflammation and improves antiviral T cell functions in HIV-infected humanized mice (30). To further evaluate whether rapamycin treatment alleviates HSC-derived CAR-T cell exhaustion and restores control over viral replication, we treated HIV-infected mice with either rapamycin or vehicle for 2 weeks at 0.5 mg/kg, 3 days a week, as described in ref. 30. In comparison with vehicle-treated mice, we observed a significant decrease in the activation markers HLA-DR and CD38, and exhaustion markers PD-1 and Tim-3, among CAR-T cells in the peripheral blood (Figure 2G). Most importantly, we observed lower viral loads in rapamycin-treated CAR mice compared with either mock or vehicle-treated CAR mice (Figure 2H). Taken together, our data suggest that rapamycin treatment alleviates CAR-T exhaustion and potentially restores the antiviral functions of CAR-T cells.

### Transcriptomic changes of CAR-T cells following rapamycin treatment in HIV-infected humanized mice.

**Figure 3:**
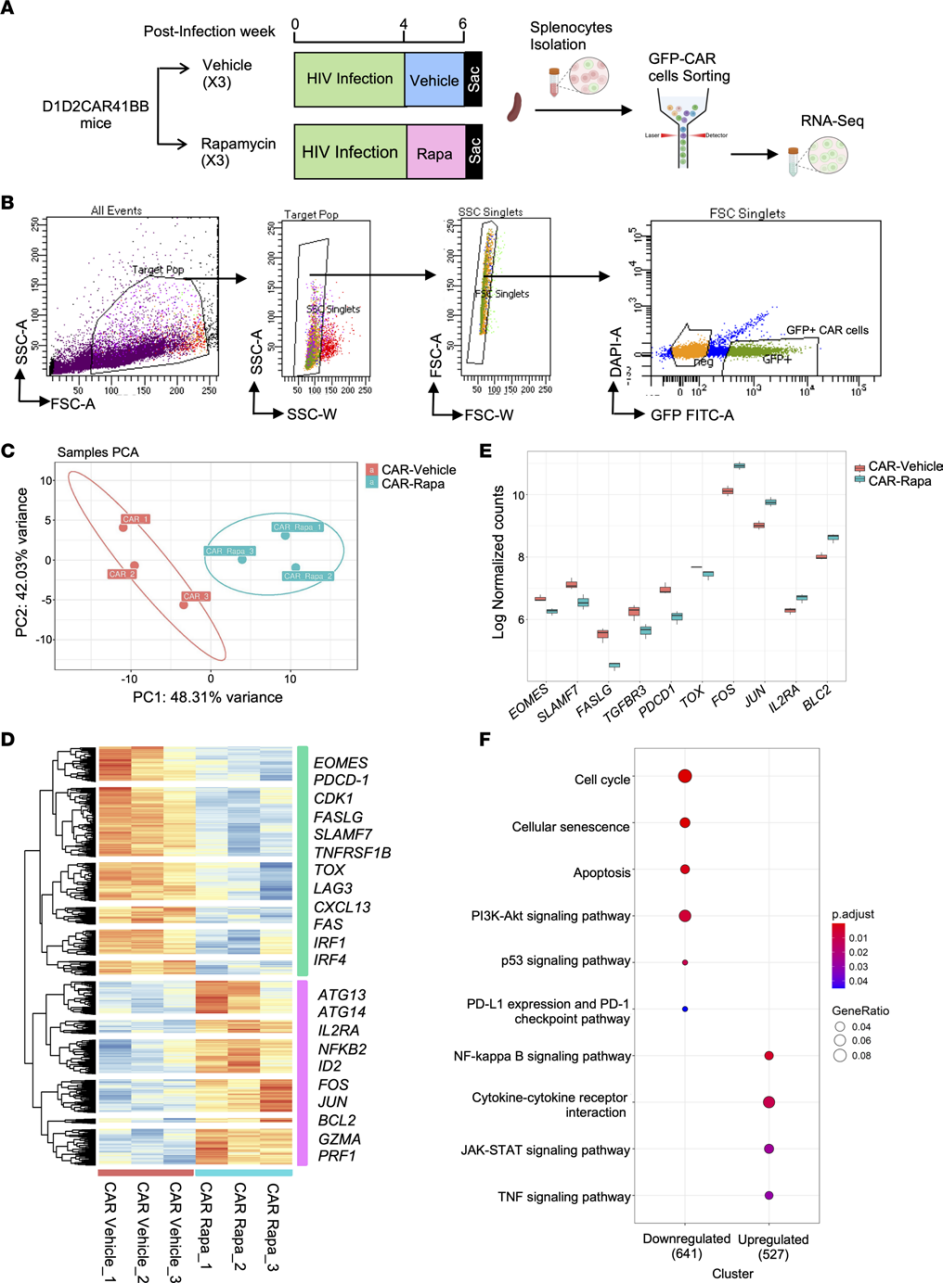
Transcriptional signatures show reduced exhaustion markers and upregulation of memory- and survival-related signaling in CAR-T cells from rapamycin-treated mice. (**A**) Humanized NSG-BLT mice with D1D2CAR41BB-modified HSCs were treated with rapamycin or vehicle for 2 weeks before necropsy. Afterward, splenocytes were isolated, GFP^+^ CAR cells were sorted, and bulk RNA sequencing was performed (*n* = 3 per group). (**B**) Representative flow cytometry analysis showing the gating strategy for sorting of GFP^+^ CAR single cells. (**C** and **D**) Principal component analysis (PCA) (**C**) and heatmap (**D**) showing the relative expression (*z* score) of the top 5,000 genes that were differentially expressed between the 2 populations of CAR-T cells derived from rapamycin-treated versus vehicle-treated CAR mice. Genes were divided into downregulated (green) and upregulated (pink) clusters by *k*-means clustering based on expression. (**E**) Box plot of log-normalized counts of genes important in T cell survival, activation, and exhaustion. Box plots show the interquartile range, median (line), and minimum and maximum (whiskers). (**F**) KEGG pathway analysis of differentially expressed genes among CAR-T cells between rapamycin-treated and vehicle-treated CAR mice. “GeneRatio” is the percentage of total DEGs in the given Gene Ontology term.

It is evident that rapamycin treatment changes the metabolic and functional activities of T cells in vivo. To more closely examine the effects of rapamycin treatment and how it can affect the CAR-T cell transcriptome, HIV-infected CAR-HSC humanized BLT mice were treated with either rapamycin or vehicle for 2 weeks before necropsy. Afterward, splenocytes were isolated, CAR^+^ cells were sorted (based on GFP expression), and bulk RNA sequencing was performed (schematically shown in Figure 3A). A total of 0.5 million to 1 million CAR (GFP^+^) cells were sorted from each mouse as shown in Figure 3B. Principal component analysis showed that CAR cells derived from untreated mice clustered separately from CAR cells isolated from rapamycin-treated mice (Figure 3C). Notably, as shown in Figure 3D, the heatmap and corresponding dendrogram clusters highlighted a downregulation of exhaustion-related markers, including the inhibitory receptors *PDCD1* (PD-1), *HAVCR2* (Tim-3), *LAG3*, and *SLAM7* and the exhaustion transcription factors *EOMES* and *TOX* (33–38), in the rapamycin-treated CAR cells. Furthermore, rapamycin induced a decrease in type I IFN–related genes, such as *CXCL13*, IFN regulatory factor 1 (*IRF1*), and *IRF4*, which are known contributors to T cell exhaustion (39–42). Corroborating our cytometric analysis (Figure 2G), these changes suggest that rapamycin mitigates T cell exhaustion.

Intriguingly, rapamycin treatment also led to an upregulation of genes associated with T cell survival and persistence, notably those of the activator protein-1 (AP-1) family, such as *JUN* and *FOS* (43–45), as shown in Figure 3D. As rapamycin is a potent autophagy inducer (30, 46), autophagy-related genes such as *ATG13* and *ATG14* were also elevated after treatment. Moreover, genes such as *IL2R*, *GZMA*, and *PRF1*, encoding IL-2 receptor, granzyme A, and perforin, respectively, which are crucial for the cytolytic activity of T cells, displayed increased expression in the rapamycin-treated CAR cells (Figure 3D). The box plot visualization revealed notable differences in the log-normalized counts of key genes between the 2 conditions as shown in Figure 3E.

We further investigated the gene expression involved in biological pathway signaling using Kyoto Encyclopedia of Genes and Genomes (KEGG) pathway analysis, shown in Figure 3F. Downstream targets of *MTOR*, including several cyclin-dependent kinases (*CDK*s) that are involved in cell cycling pathways, were reduced. Concurrently, cell senescence and apoptotic pathways were downregulated, as shown by lower *FAS* signaling and increased expression of the anti-apoptotic gene *BCL2* in rapamycin-treated CAR-T cells (Figure 3, E and F). At the same time, *NF-kB*, *JAK-STAT*, and *TNF* signaling pathways were upregulated in CAR-T cells from rapamycin-treated mice. Collectively, these findings underscore rapamycin’s comprehensive role in modulating CAR-T cell function, effectively reducing exhaustion markers, and enhancing both survival and cytotoxic capabilities.

### Low-dose rapamycin treatment in combination with ART reduced exhaustion of CAR-T cells and significantly reduced viral rebound.

**Figure 4:**
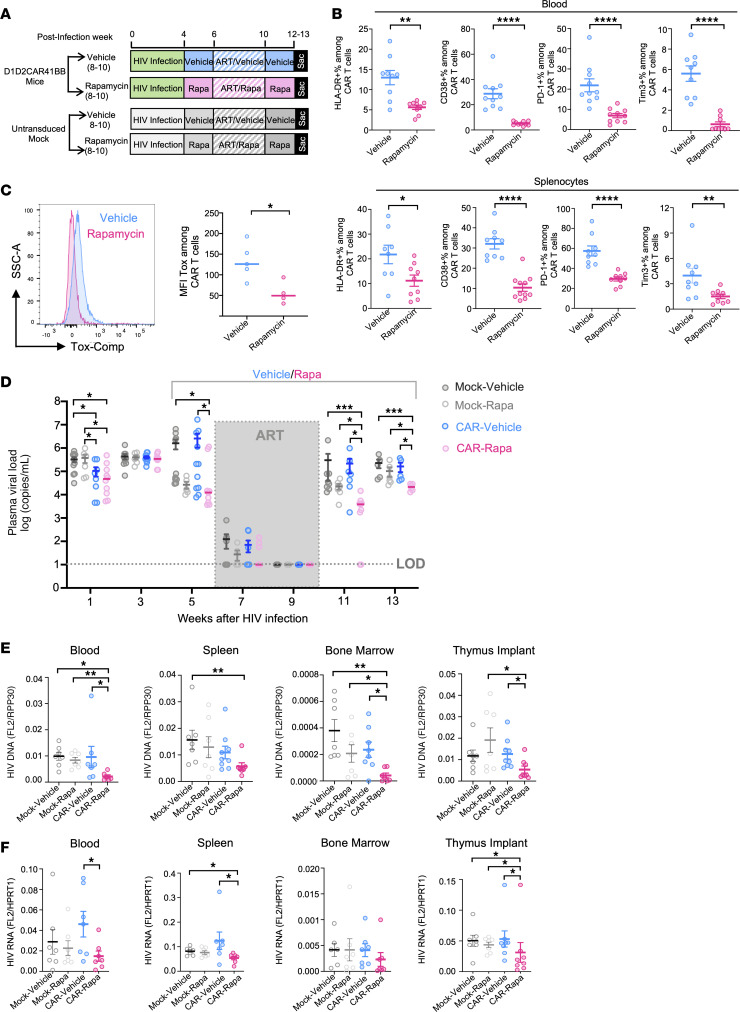
Long-term low-dose rapamycin treatment in combination with ART alleviates CAR-T cell exhaustion and reduces viral rebound. (**A**) Humanized NSG-BLT mice with either D1D2CAR41BB-modified or nonmodified HSCs were infected with HIV and treated with rapamycin or vehicle for 2 weeks. Afterward, while rapamycin or vehicle treatment was continued, mice were treated with ART for 4 weeks, followed by ART interruption for 2–3 weeks. (**B**) PD-1, Tim-3, HLA-DR, and CD38 expression was measured by flow cytometry (quantified by gating of percentage positive cells) on peripheral blood (top) or spleen (bottom) CD3^+^CAR^+^ T cells (*n* = 8–10 per group). (**C**) Splenocytes from CAR mice treated with ART and rapamycin or vehicle were isolated and stained with intracellular antibodies against human TOX1. MFIs of the TOX1 on CAR^+^ T cells were measured by flow cytometry (*n* = 5 per group). (**D**) Longitudinal HIV viral load in plasma from humanized mice after rapamycin or vehicle treatment was measured by real-time PCR (*n* = 4–7 per group). Dotted line indicates limit of detection. (**E**) HIV DNA copies per cell from PBMCs, splenocytes, bone marrow, or thymus implant from different groups of mice as measured by real-time PCR. Human RPP30 gene was used as internal control (*n* = 7–9 per group). (**F**) Relative HIV cellular RNA expression from multiple lymphoid tissues from different groups of mice as measured by real-time PCR. Human HPRT1 gene expression was used as internal control (*n* = 7–8 per group). The Mann-Whitney test was used to compare 2 groups, and the Kruskal-Wallis test was used for multiple comparisons (**D**–**F**). *P < 0.05, **P < 0.01, ***P < 0.001, ****P < 0.0001.

To further assess the effects of rapamycin treatment in combination with ART, we treated HIV-infected humanized mice transplanted with mock or CAR-modified HSCs with rapamycin or vehicle for 2 weeks, followed by 4 weeks of ART treatment and ART withdrawal (Figure 4A). As shown in Figure 4B, in comparison with control mice, ART combined with rapamycin significantly reduced the expression of immune activation markers (HLA-DR, CD38) and exhaustion markers (PD-1, Tim-3) among CAR-T cells in the blood and spleen at necropsy. TOX is a key transcription factor that regulates the T cell exhaustion program, and the expression of TOX has been associated with cellular exhaustion during chronic infection (33, 35, 47). As in our RNA sequencing studies (Figure 3D), flow analysis of CAR-T cells at necropsy also showed significant downregulation of TOX expression of CAR-T cells from mice treated with rapamycin as compared with vehicle control (Figure 4C).

To study the effects of rapamycin treatment on HIV suppression, persistence, and viral rebound in CAR mice, plasma viral loads were measured longitudinally. As shown in Figure 4D, at 9 weeks after HIV infection and after 4 weeks of ART, all mice exhibited full viral suppression with undetectable viral load. One week after interruption of ART, all mock control and vehicle-treated CAR mice and all but 2 rapamycin-treated CAR mice experienced viral rebound. Three weeks after ART withdrawal, all mice showed viral rebound. However, rapamycin-treated CAR mice maintained a significantly lower viral load as compared with vehicle-treated CAR mice, or mock mice treated with either vehicle or rapamycin. Additionally, we observed significantly lower levels of viral DNA (Figure 4E) and cell-associated HIV RNA (Figure 4F) in the blood, spleen, and bone marrow at necropsy after ART withdrawal, indicating a reduction in overall viral burden in the rapamycin-treated CAR mice. Taken together, these data suggest that the combination of rapamycin and ART treatment improves CAR-T cell function in HIV suppression and reduces viral rebound.

### Rapamycin treatment reduces mitochondrial ROS in CAR-T cells and improves CAR-T function.

**Figure 5:**
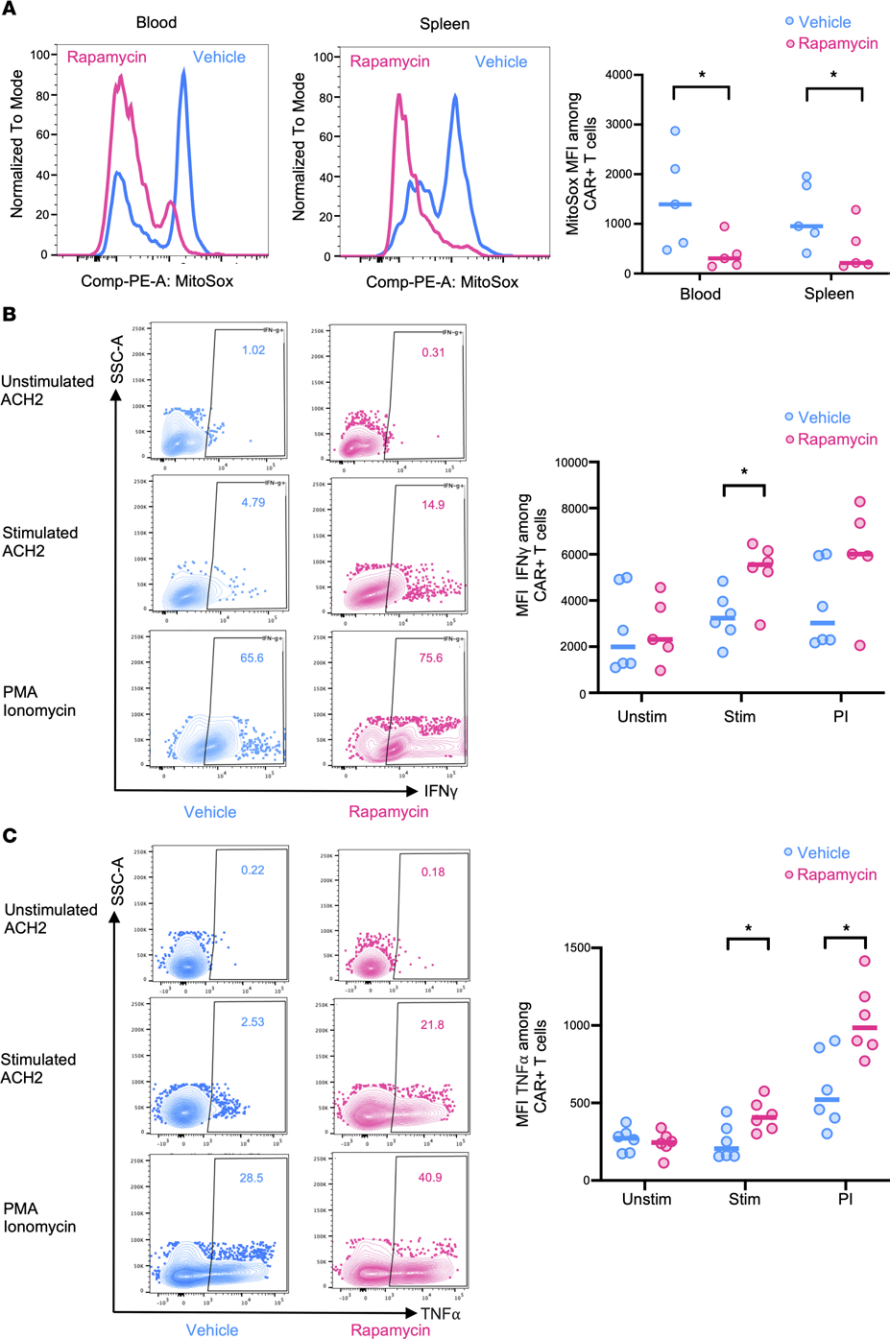
Rapamycin treatment decreases mitochondrial ROS and improves CAR-T function in vivo. (**A**) ROS levels analyzed by MitoSOX staining in blood or spleen CAR-T cells isolated from CAR mice treated with either rapamycin or vehicle (*n* = 5 per group). (**B** and **C**) Splenocytes from CAR-transduced, HIV-1–infected, vehicle-treated, or rapamycin-treated mice were stimulated with PMA/ionomycin (PI) or Env-expressing (stimulated ACH2) or non-Env-expressing (unstimulated ACH2) cells, and production of IFN-γ and TNF-α by CAR-T cells was measured by flow cytometry (*n* = 5–6 per group). Representative flow cytometry data showing percentage and MFI of IFN-γ^+^ (**B**) and TNF-α^+^ (**C**) among CAR-T cells from HIV-1–infected, vehicle-treated, or rapamycin-treated mice. Each dot represents an individual mouse; horizontal bars indicate median values. Mann-Whitney test (unpaired); *P < 0.05.

Excessive ROS can cause damage to cellular components, including lipids, proteins, and DNA (48, 49). Decreased mitochondrial biogenesis and excessive production of mitochondrial ROS can exacerbate mitochondrial dysfunction and immune exhaustion in T cells (50–54). Our in vitro data suggested that rapamycin treatment protects the mitochondria against oxidative stress in CAR-T cells (Figure 1D). To further investigate the potential for in vivo rapamycin treatment to reduce oxidative stress and alleviate mitochondrial injuries in CAR-T cells, we measured mitochondrial dysfunction levels using MitoSOX to detect mitochondrial superoxide levels in CAR-T cells from both peripheral blood and spleen of CAR mice treated with rapamycin or vehicle control. We observed that CAR-T cells from mice treated with rapamycin exhibited significantly lower mitochondrial ROS compared with vehicle-treated mice (Figure 5A), suggesting a reduction of oxidative stress in treated mice.

To further investigate whether HIV-specific CAR-T cell responses were improved in the ART and rapamycin combined-treatment group, splenocytes from rapamycin- or vehicle-treated mice were stimulated with the mitogens PMA and ionomycin, or stimulated with HIV target cells (stimulated latently infected ACH2 cells, which are Env^+^) or control cells (unstimulated ACH2 cells, which are Env^–^). Compared with the vehicle-treated control, CAR-T cells from rapamycin-treated infected mice produced significantly higher levels of proinflammatory IFN-γ and TNF-α cytokines after PMA/ionomycin stimulation (Figure 5, B and C), and HIV Env^+^ cells, indicating improved proinflammatory cytokine production and antiviral responses of CAR-T cells. In summary, these data suggest that a combination of rapamycin and ART reduced ROS and improved anti-HIV functions of CAR-T cells in vivo.

### Short-term rapamycin treatment in CAR-HSC NSG-Tg(IL-15) humanized mice showed delayed viral rebound and smaller reservoirs after ART withdrawal.

**Figure 6:**
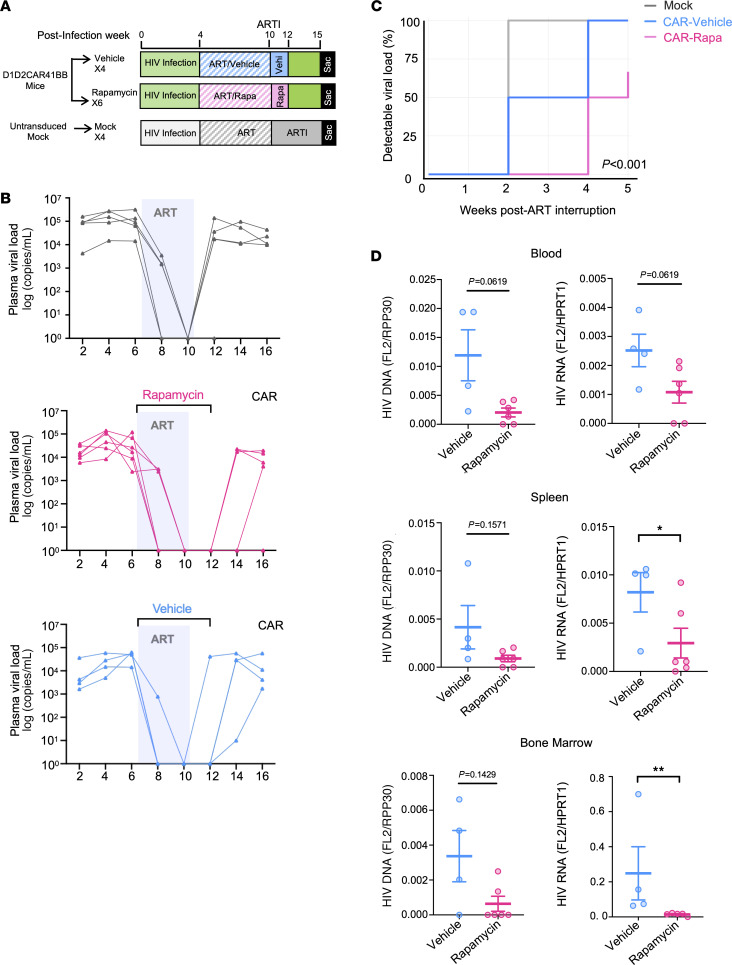
Rapamycin-treated NSG-IL-15 CAR mice show significantly delayed viral rebound and smaller reservoirs after ART withdrawal. (**A**) Humanized NSG-IL-15-BLT mice were constructed with either unmodified HSCs or HSCs modified with D1D2CAR41BB. After immune reconstitution, mice were infected with HIV-1_NFNSXL9_. Four weeks after infection, mock mice were treated with ART only. Mice with CAR-modified HSCs were treated with rapamycin or vehicle along with ART. After successful viral load suppression, ART was interrupted, and rapamycin or vehicle treatment continued for 2 additional weeks before discontinuation (*n* = 4–6 per group). (**B**) Longitudinal plasma HIV viral load as measured by real-time PCR. (**C**) Survival analysis of time to detectable viral load among mock mice and CAR mice that were treated with vehicle or rapamycin. *P* < 0.0001 by log-rank test. (**D**) HIV DNA and relative cellular HIV RNA expression from blood PBMCs, splenocytes, and bone marrow as measured by real-time PCR. Mann-Whitney test (unpaired); *P < 0.05, **P < 0.01.

An enhanced humanized mouse model, termed Hu-NSG-Tg(IL-15), which was engineered to express a physiological level of human IL-15, was reported to support more robust engraftment of human immune cells, including T cells, B cells, and NK cells, and therefore represents a valuable model for studying HIV pathogenesis and immune responses (55, 56). We adopted this new model to produce CAR-HSC mice and examined the effects of rapamycin or vehicle treatment. Importantly, to examine whether the effects of rapamycin treatment persist after short-term treatment, rapamycin treatment was started with ART and stopped 2 weeks after ART withdrawal, and mice were continuously monitored for an additional 3 weeks before necropsy (Figure 6A). As shown in Figure 6B, 2 weeks after ART interruption, viral loads quickly rebounded in all mice (100%, 4/4) from the mock group. In the CAR-HSC vehicle-treated group, 2 of 4 did not rebound (50%) 2 weeks after ART withdrawal. However, all mice rebounded 4 weeks after ART interruption. In contrast, none of the rapamycin-treated CAR-HSC mice had viral rebound 2 weeks after ART. Two weeks after cessation of rapamycin treatment and 4 weeks after ART cessation, 3 of 6 (50%) rapamycin-treated CAR-HSC mice did not rebound. Five weeks after ART withdrawal at necropsy, 2 of 6 (33%) rapamycin-treated CAR-HSC mice continued to have undetectable viral load, while the other 4 maintained lower viral loads after rebound. In summary, we observed that rapamycin-treated CAR mice had improved CAR suppression of viral replication, leading to delayed viral rebound after ART cessation, as shown in Figure 6C. Importantly, as shown in Figure 6D, we observed an overall decrease in the level of cell-associated HIV DNA and RNA in the spleen and bone marrow in rapamycin-treated CAR mice compared with vehicle-treated CAR mice, and mice with undetectable viral loads also showed low/undetectable viral DNA/RNA in tissues, suggesting lower reservoir in rapamycin-treated CAR mice.

## Discussion

CAR-redirected T cell immunity against HIV-1 represents a highly promising approach that can be used in most HIV-infected individuals. CARs recognize target cells through direct binding to specific cell surface antigens and are HLA-unrestricted, bypassing a major limitation for T cell immunotherapies (57). Though they are now widely applied for cancer, some of the first clinical trials of CARs were for HIV-1 infection (58–60). The “original” HIV-specific CARs were composed of a CD4 extracellular domain linked to the intracellular CD3-ζ signaling domain (CD4CAR), using CD4 binding to HIV-1 Env for targeting and killing of HIV-1–infected cells (8). HIV-1 Env interaction with CD4 is critical for viral replication, thus limiting viral escape from a CD4-based CAR (61). Tremendous progress has been made using CD4-based CAR-T cell therapy against HIV infection since its invention. Improvements in the CAR design have been made with modification of the Env recognition domain and inclusion of costimulatory domains, and enhanced CD4-based CAR-T cell efficacy has been demonstrated by multiple groups of investigators (6, 15, 62–64), including ours (10, 12, 13, 65–68); CD4-based CAR-T cell therapy is currently under investigation in multiple clinical trials (ClinicalTrials.gov NCT03617198, NCT04648046).

Given the challenge of HIV latent reservoirs driving chronic infection and persistence under suppressive ART, the functional persistence of CAR-T cells is critical for successful long-term immune containment of HIV infection. HSC-based gene therapy supports lifelong generation of functional immune progeny, giving rise to a stable supply of gene-modified CAR-T cells. We have shown that HSC-based CAR therapy allows for lifelong, persistent production of functional CAR-T cells to control viral replication from reactivated reservoirs, and HSC-based CAR therapy showed better persistence and trafficking to tissue reservoirs than peripheral blood–derived CAR-T cells (10, 11, 13, 65, 67). Despite their in vivo efficacy in reducing viral replication and reservoir, HSC-derived CAR-T cells cannot achieve full viral suppression after ART withdrawal. Persistent type I IFN signaling and immune activation during chronic HIV infection are driving forces of immune dysfunction (69, 70), and engineered CAR-T cells are also subject to this immune exhaustion. Previously we demonstrated that targeting persistent type I IFN signaling, either by blocking type I IFN receptor (71) or by inducing autophagy with rapamycin (30, 69, 72), can lead to reduced hyperinflammation and improved anti-HIV T cell function in vivo. In the current study, we found that rapamycin treatment also improved CAR-HSC therapy in HIV-infected humanized mice. Rapamycin treatment reduced persistent immune activation and rejuvenated CAR-T cell function, leading to delayed viral rebound and better viral control after ART cessation, even after rapamycin treatment was stopped. Notably, 2 of 6 rapamycin-treated CAR-HSC mice continued to have undetectable viral loads 5 weeks after ART withdrawal and 3 weeks after rapamycin cessation, while all other groups had 100% viral rebound. The rapamycin treatment also led to reduced cell-associated HIV RNA and DNA in blood and multiple lymphoid tissues in CAR-HSC mice. Importantly, we observed improved mitochondria function and substantial transcriptomic modification of CAR-T cells by rapamycin treatment, suggesting the dual beneficial effects of rapamycin in reducing T cell inhibitory receptors while simultaneously promoting stemness-related gene expression, offering promising insights into the optimization of CAR-T cell therapy for sustained anti-HIV responses.

Rapamycin inhibits mTOR, a major regulator of cellular metabolism and the cellular aging process. First discovered and FDA-approved for treatment of various cancers and as an immunosuppressant, the effects of rapamycin on aging are increasingly recognized and used for longevity studies (20, 73). Multiple studies suggest that rapamycin treatment extends health span and improves the function of the aging immune system, such as improving antiviral activities in older adults (21, 22, 28). This is relevant to HIV, as PLWH are aging with greater life expectancy due to effective ART. Despite the success of ART, the difference in comorbidity-free years between PLWH and the general population persists, and premature immune aging has been shown to be the major culprit driving age-related comorbidity (74–76). A recent clinical study demonstrated that sirolimus (rapamycin) reduced CD4^+^ T cell cycling and PD-1 expression on CD8^+^ T cells in PLWH, underscoring its potential to impact HIV reservoirs by modulating immune activation and exhaustion pathways (77). These findings align with our observations in humanized mice and support previous findings where treatment with rapamycin was correlated with reduced HIV reservoir in HIV-1–infected kidney transplant recipients (78). Rapamycin also has documented antiviral properties through mTOR inhibition (79). To further delineate the dual mechanisms of rapamycin — direct antiviral effects and immunomodulation (80) — additional studies such as depleting CD8^+^ T cell responses could help clarify the role of rapamycin in modulating immune-mediated clearance of infected cells versus its direct impact on viral replication. In addition, while our study highlights the effects of rapamycin in enhancing CAR-T cell function, its potential to directly modulate endogenous CTLs and other immune cell populations, such as NK cells, should also be considered. Therefore, additional studies should be carried out to further explore the therapeutic effects of rapamycin on different immune cell types. However, as a master regulator, mTOR signaling plays a key role in T cell fate, such as effector versus memory differentiation, and its regulation needs to be tightly controlled (81). Treatment with daily rapamycin can limit T cell proliferation in SIV-infected rhesus macaques on antiretroviral therapy and was not shown to impact SIV reservoir (82). For our current study, we chose to use lower and intermittent dosing of rapamycin that we previously described, which did not impact T cell homeostasis (30). Therefore, careful studies on the dosing and treatment regimen of rapamycin are needed to maximize its effects and reduce its toxicity in animal models and in clinical studies.

In summary, our study describes the effects of rapamycin to improve the function of anti-HIV CAR-T cells in vivo, demonstrating its impact on CAR-T cell metabolism and transcriptomic modification. We believe that the results described in this study shed light on potential strategies to augment CAR-T functions for treating HIV infection, and our findings may also be applicable to other CAR-T cell therapies that are affected by immune exhaustion (83).

## Methods

### Sex as a biological variable.

Both male and female human PBMCs or tissue donors and animals were included in all experiments.

### Lentivirus production.

The lentivirus-based D1D2CAR41BB vectors were produced in Lenti-X 293T cells (Takara Bio) using the Lipofectamine 2000 reagent (Invitrogen). Briefly, Lenti-X 293T cells were cotransfected with D1D2CAR41BB vector with pCMV.ΔR8.2.Δvpr packaging construct and the pCMV-VSV-G envelope protein plasmid, as previously described (1, 2). The supernatant was obtained from transfected Lenti-X 293T cells 48 hours after transfection. It underwent filtration using a 0.45 μm sterile filter and concentration through ultracentrifugation using a Beckman SW32 rotor at 154,000*g* at 4°C. After aspiration of the medium, the pellet was resuspended in PBS and stored at –80°C.

### Transduction of CAR-T cells.

Primary T cells were sorted from primary PBMCs from healthy donors using a Pan T Cell Isolation Kit, human (Miltenyi Biotec, 130-096-535). Isolated T cells were stimulated with plate-bound anti-CD3 and anti-CD28 (Miltenyi Biotec) at 2 million cells/mL. After activation for at least 24 hours, cells were washed and transduced with CD4CAR vector on a retronectin-coated plate with cytokine IL-2. Cells were cultured in RPMI supplemented with 10% FBS and 1% penicillin/streptomycin with IL-2 for additional 2 weeks. After this culture period, the cells were treated with either DMSO control or rapamycin at a concentration of 500 pM for 2 days before the Seahorse assay was conducted.

### Seahorse assay.

CAR-T cells (total 250,000) were seeded into wells of a poly-d-lysine–coated (100 μg/mL) XF96 spheroid plate (Agilent Technologies). Cells in the plate were centrifuged at 200*g* for 7 minutes with no centrifuge brake, and mitochondrial respiration was measured using the Seahorse XF96 extracellular flux analyzer equipped with a spheroid plate–compatible thermal tray (Agilent Technologies). Basal respiration was first measured in 3 mM glucose medium. To validate cell respirometry with the XF96 spheroid plate, CAR-T cells were then sequentially exposed to glucose (final concentration in well of 20 mM), oligomycin A (3.5–4.5 μM final concentration), FCCP (1 μM final concentration), and antimycin A (2.5 μM final concentration).

### Generation of humanized mice.

D1D2CAR41BB BLT mice were constructed similarly to previously reported HIV-1 triple CAR BLT humanized mice (68). Briefly, human fetal liver–derived CD34^+^ cells were purified by immunomagnetic separation. Cells were then transduced overnight with D1D2CAR41BB lentiviruses with retronectin-coated plates. On the day of transplant, NOD.Cg-*Prkdc^scid^ Il2rg^tm1Wjl^*/SzJ (NOD/SCID/IL2Rγ^−/−^, or NSG, The Jackson Laboratory) or NOD.Cg-*Prkdc^scid^* *Il2rg^tm1Wjl^* Tg(IL15)1Sz/SzJ (NSG-huIL15, The Jackson Laboratory) mice received 2.7 Gy total-body sublethal irradiation and then underwent transplantation of transduced CD34^+^ cells in Matrigel (Corning Life Sciences) and liver and thymus tissue under the kidney capsule; the tissue was from the same donor as the CD34^+^ cells. Afterward, mice were injected with approximately 0.5 × 10^6^ lentivirus-based CAR vector–transduced CD34^+^ cells. At 8–10 weeks after transplantation, each mouse was bled retro-orbitally, and PBMCs were analyzed by flow cytometry to check human immune cell engraftment. Upon stable human leukocyte reconstitution efficiency greater than 50%, mice were used for HIV-1 infection and further experiments.

### HIV-1 infection, ART, and rapamycin treatment.

The R5 tropic strain of HIV-1_NFNSXSL9_ was generated by transfection of 293T cells with plasmid containing full-length HIV-1_NFNSXSL9_ genome. Humanized mice were infected with HIV-1_NFNSXSL9_ (500 ng p24 per mouse) through retro-orbital injection while under inhalant general anesthesia. Infected mice with demonstrable viral infection were treated for 6 weeks with ART drugs. The ART regimen consisted of tenofovir disoproxil fumarate (TDF; 80 mg/kg), emtricitabine (FTC; 120 mg/kg), and elvitegravir (ELV; 160 mg/kg) given by food. TDF, FTC, and ELV were supplied by Gilead Sciences. TDF, FTC, and ELV were dissolved in DMSO and mixed with sweetened moist gel meal (DietGel Boost, ClearH_2_O; Medidrop Sucralose) as previously described (84). For rapamycin treatment, mice were injected i.p. with 0.5 mg/kg rapamycin (LC Laboratories) 3 times a week.

### Flow cytometry.

Mitochondria-associated ROS levels were measured by staining of cells with MitoSOX (Molecular Probes/Invitrogen) at 5 μM for 40 minutes at 37°C. Cells were then washed with PBS solution and resuspended in PBS solution containing 2% FBS for FACS analysis. Single-cell suspensions prepared from peripheral blood or spleen of humanized mice were stained using the following antibodies for flow cytometry: CD45 (Invitrogen, clone HI30), CD3 (Invitrogen, clone OKT3), CD4 (Invitrogen, clone RPA-T4), CD8 (Invitrogen, clone SK1), CD38 (Invitrogen, clone HIT2), HLA-DR (BD Biosciences, clone L240), CD45RA (BD Biosciences, clone HI100), CD62L (Invitrogen, clone DREG-56), IFN-γ (BioLegend, clone 4S.B3), IL-2 (BioLegend, clone MQ1-17H12), TNF-α (BioLegend, clone Mab11), TOX (Invitrogen, clone TXRX10), PD-1 (Invitrogen, clone ebioJ105), and Tim-3 (BioLegend, clone F38-2E2). LIVE/DEAD Fixable Yellow Dead Cell Stain Kit (Invitrogen) was used. Antibodies for cell surface markers and intracellular markers were conjugated to FITC, phycoerythrin (PE), PerCP-Cy5.5, PE-Cy5, PE-Cy7, electron coupled dye (ECD), allophycocyanin (APC), APC–eFluor 780, Alexa Fluor 700, eFluor 450, Pacific Orange, or Pacific Blue in the appropriate combination. An LSRFortessa flow cytometer and FACSDiva software (BD Biosciences) were used to obtain the cells, while FlowJo software was used for data analysis. A minimum of 1,000 cells were acquired for each analysis, and each flow plot, representative of the data, was replicated more than 3 times.

### Nucleic acid extraction and real-time PCR.

To measure HIV plasma viremia, viral RNA was extracted from plasma and 1-step real-time PCR was performed using the TaqMan RNA-to-Ct 1-Step Kit (Thermo Fisher Scientific) with the following primers and probe: HIV-1 forward primer, 5′-CAATGGCAGCAATTTCACCA-3′; HIV-1 reverse primer, 5′-GAATGCCAAATTCCTGCTTGA-3′; HIV-1 probe, 5′-[6-FAM] CCCACCAACAGGCGGCCTTAACTG [Tamra-Q]-3′.

To measure the levels of cell-associated HIV RNA with HPRT1 as an internal control, cells were harvested for RNA extraction according to the manufacturer’s protocol (QIAGEN) and making of cDNA using the High-Capacity cDNA Reverse Transcription Kit (Thermo Fisher Scientific). For HPRT1, Single Tube TaqMan Gene Expression Assays (Thermo Fisher Scientific) human HPRT1 (Hs01003267_m1) was used. Relative mRNA expression was calculated by normalizing of genes to HPRT1 mRNA expression.

To measure the levels of cell-integrated HIV DNA with RPP30 as an internal control, cells were harvested for DNA extraction according to the manufacturer’s protocol (QIAGEN). HIV DNA and RPP30 levels were quantified using Single Tube TaqMan Gene Expression Assays (Thermo Fisher Scientific) following the manufacturer’s protocol. Relative DNA integration was calculated by normalizing HIV DNA levels to RPP30 gene copy numbers, serving as an endogenous control. The following primers and probes were used for quantitative PCR: HIV-1 forward primer, 5′-CAATGGCAGCAATTTCACCA-3′; HIV-1 reverse primer, 5′-GAATGCCAAATTCCTGCTTGA-3′; HIV-1 probe, 5′-[6-FAM] CCCACCAACAGGCGGCCTTAACTG [Tamra-Q]-3′; RPP30 forward primer, 5′-GATTTGGACCTGCGAGCG-3′; RPP30 reverse primer, 5′-GCGGCTGTCTCCACAAGT-3′; RPP30 probe, /5HEX/TTCTGACCT/ZEN/GAAGGCTCTGCG/3IABkFQ/-3′.

### D1D2CAR sorting and RNA sequencing.

Spleens from D1D2CAR-transduced mice were collected, mashed over a 70 μm cell strainer, and resuspended into single-cell suspensions in complete RPMI after red blood cell lysis using ACK lysis buffer (Thermo Fisher Scientific). A total of 0.5 million to 1 million GFP^+^ single live cells per mouse were sorted from splenocytes using BD FACS Melody (BD Biosciences). Three replicates of each experiment were carried out. RNA extraction was done using RNeasy kits (QIAGEN). Sample quality control and integrity (RNA integrity number–equivalent values) were performed using Tapestation Analysis software v3.2 (Agilent Technologies). Sequencing was carried out using the Illumina NovaSeq platform. Raw sequence data of different treatment conditions (in triplicate) were pre-processed for quality using FastQC (Babraham Institute; http://www.bioinformatics.babraham.ac.uk/projects/fastqc). Trimmomatic was used for adapters and quality trimming (85). After this, reads were aligned onto human genome (hg38) using STAR aligner (86). SAMtools was used to convert SAM files to BAM files (87). Mapped reads were counted across human genes using the tool featureCounts (75), which provided raw count data by assigning mapped reads to genes. Differential gene expression analysis was performed on the raw read count data using the R package DESeq2. Raw sequence data and processed data were deposited to GEO. Gene expression data were analyzed using the Gene Set Enrichment Analysis software tool (88). For pathway analysis, STRING (Search Tool for the Retrieval of Interacting Genes/Proteins) software was used (89).

### Statistics.

A total of 2 independent cohorts of humanized mice were generated. Each cohort was generated from cells derived from the same donor tissues (*n* = 15–30 total mice per cohort). Two cohorts were pooled for comparing between groups. All statistical analyses were performed using Prism 9.0 software (GraphPad) or R software v4.3.1. To compare statistical difference between 2 groups, Mann-Whitney *U* tests were used. For analysis of data that contained more than 2 groups (depicted in Figure 2, B–E and H, and Figure 4, D–F), the Kruskal-Wallis test was performed to compare samples. *P* values less than 0.05 by Kruskal-Wallis or Mann-Whitney test were considered statistically significant. Specific statistical tests are also reported in the figure legends.

### Study approval.

PBMCs were obtained at UCLA in accordance with UCLA IRB–approved protocols under written informed consent using an IRB-approved written consent form by the UCLA Center for AIDS Research Virology Laboratory and distributed for this study without personal identifying information. Human fetal tissue was purchased from Advanced Bioscience Resources or Cercle Allocation Services and was obtained without identifying information and did not require IRB approval for its use. Animal research described in this article was performed under the written approval of the UCLA Animal Research Committee in accordance with all federal, state, and local guidelines. All surgeries were performed under ketamine/xylazine and isoflurane anesthesia, and all efforts were made to minimize animal pain and discomfort.

### Data availability.

Raw data are in the Supporting Data Values file. RNA-sequencing data generated in this study were deposited in the NCBI’s Gene Expression Omnibus database (accession GSE284491). No original code is reported.

## Author contributions

WM and AZ designed the experiments. WM, ST, JH, NK, VR, EC, VP, MC, HM, HN, and LW conducted the experiments. WM, JH, ST, NK, and AZ analyzed the data. WM and AZ wrote the original draft. JH, ST, NK, HM, MDM, and SGK reviewed and edited the manuscript.

## Supplementary Material

Supplemental data

Supporting data values

## Version 1 02/11/2025

In-Press Preview

## Version 2 04/01/2025

Electronic publication

## References

[1] Collins DR (2020). CD8^+^ T cells in HIV control, cure and prevention. Nat Rev Immunol.

[2] Perdomo-Celis F (2019). CD8^+^ T-cell response to HIV infection in the era of antiretroviral therapy. Front Immunol.

[3] Deng K (2015). Broad CTL response is required to clear latent HIV-1 due to dominance of escape mutations. Nature.

[4] Blackburn SD (2009). Coregulation of CD8+ T cell exhaustion by multiple inhibitory receptors during chronic viral infection. Nat Immunol.

[5] Marsden MD, Zack JA (2015). Double trouble: HIV latency and CTL escape. Cell Host Microbe.

[6] Mu W (2020). Engineering CAR T cells to target the HIV Reservoir. Front Cell Infect Microbiol.

[7] Louie RHY (2018). Fitness landscape of the human immunodeficiency virus envelope protein that is targeted by antibodies. Proc Natl Acad Sci U S A.

[8] Yang OO (1997). Lysis of HIV-1-infected cells and inhibition of viral replication by universal receptor T cells. Proc Natl Acad Sci U S A.

[9] Rafiq S (2020). Engineering strategies to overcome the current roadblocks in CAR T cell therapy. Nat Rev Clin Oncol.

[10] Zhen A (2021). Robust CAR-T memory formation and function via hematopoietic stem cell delivery. PLoS Pathog.

[11] Zhen A (2015). HIV-specific immunity derived from chimeric antigen receptor-engineered stem cells. Mol Ther.

[12] Zhen A (2017). Long-term persistence and function of hematopoietic stem cell-derived chimeric antigen receptor T cells in a nonhuman primate model of HIV/AIDS. PLoS Pathog.

[13] Carrillo MA (2024). Stem cell-derived CAR T cells show greater persistence, trafficking, and viral control compared to ex vivo transduced CAR T cells. Mol Ther.

[14] Pauken KE, Wherry EJ (2015). Overcoming T cell exhaustion in infection and cancer. Trends Immunol.

[15] Maldini CR (2020). Dual CD4-based CAR T cells with distinct costimulatory domains mitigate HIV pathogenesis in vivo. Nat Med.

[16] Rust BJ (2020). Robust expansion of HIV CAR T cells following antigen boosting in ART-suppressed nonhuman primates. Blood.

[17] Grosser R (2019). Combination immunotherapy with CAR T cells and checkpoint blockade for the treatment of solid tumors. Cancer Cell.

[18] Martins F (2019). Adverse effects of immune-checkpoint inhibitors: epidemiology, management and surveillance. Nat Rev Clin Oncol.

[19] Bajwa R (2019). Adverse effects of immune checkpoint inhibitors (programmed death-1 inhibitors and cytotoxic T-lymphocyte-associated protein-4 inhibitors): results of a retrospective study. J Clin Med Res.

[20] Mannick JB, Lamming DW (2023). Targeting the biology of aging with mTOR inhibitors. Nat Aging.

[21] Mannick JB (2014). mTOR inhibition improves immune function in the elderly. Sci Transl Med.

[22] Mannick JB (2021). Targeting the biology of ageing with mTOR inhibitors to improve immune function in older adults: phase 2b and phase 3 randomised trials. Lancet Healthy Longev.

[23] Di Benedetto F (2010). First report on a series of HIV patients undergoing rapamycin monotherapy after liver transplantation. Transplantation.

[24] Henrich TJ (2021). Everolimus, an mTORC1/2 inhibitor, in ART-suppressed individuals who received solid organ transplantation: a prospective study. Am J Transplant.

[25] Wirth M (2019). Effects of spermidine supplementation on cognition and biomarkers in older adults with subjective cognitive decline (SmartAge)—study protocol for a randomized controlled trial. Alzheimers Res Ther.

[26] Pollizzi KN (2015). mTORC1 and mTORC2 selectively regulate CD8+ T cell differentiation. J Clin Invest.

[27] Pearce EL (2009). Enhancing CD8 T-cell memory by modulating fatty acid metabolism. Nature.

[28] Mannick JB (2018). TORC1 inhibition enhances immune function and reduces infections in the elderly. Sci Transl Med.

[29] Ando S (2023). mTOR regulates T cell exhaustion and PD-1-targeted immunotherapy response during chronic viral infection. J Clin Invest.

[30] Mu W (2022). Autophagy inducer rapamycin treatment reduces IFN-I-mediated inflammation and improves anti-HIV-1 T cell response in vivo. JCI Insight.

[31] McLane LM (2019). CD8 T cell exhaustion during chronic viral infection and cancer. Annu Rev Immunol.

[32] Hashimoto M (2018). CD8 T cell exhaustion in chronic infection and cancer: opportunities for interventions. Annu Rev Med.

[33] Alfei F (2019). TOX reinforces the phenotype and longevity of exhausted T cells in chronic viral infection. Nature.

[34] Scott AC (2019). TOX is a critical regulator of tumour-specific T cell differentiation. Nature.

[35] Khan O (2019). TOX transcriptionally and epigenetically programs CD8^+^ T cell exhaustion. Nature.

[36] Buggert M (2014). T-bet and Eomes are differentially linked to the exhausted phenotype of CD8+ T cells in HIV infection. PLoS Pathog.

[37] O’Connell P (2021). SLAMF7 signaling reprograms T cells toward exhaustion in the tumor microenvironment. J Immunol.

[38] Seo W (2021). Transcriptional regulatory network for the establishment of CD8^+^ T cell exhaustion. Exp Mol Med.

[39] Tietscher S (2023). A comprehensive single-cell map of T cell exhaustion-associated immune environments in human breast cancer. Nat Commun.

[40] Sumida TS (2022). Type I interferon transcriptional network regulates expression of coinhibitory receptors in human T cells. Nat Immunol.

[41] Shao L (2019). IRF1 inhibits antitumor immunity through the upregulation of PD-L1 in the tumor cell. Cancer Immunol Res.

[42] Man K (2017). Transcription factor IRF4 promotes CD8^+^ T cell exhaustion and limits the development of memory-like T cells during chronic infection. Immunity.

[43] Seo H (2021). BATF and IRF4 cooperate to counter exhaustion in tumor-infiltrating CAR T cells. Nat Immunol.

[44] Lynn RC (2019). c-Jun overexpression in CAR T cells induces exhaustion resistance. Nature.

[45] Karin M (1997). AP-1 function and regulation. Curr Opin Cell Biol.

[46] Kim YC, Guan KL (2015). mTOR: a pharmacologic target for autophagy regulation. J Clin Invest.

[47] Yao C (2019). Single-cell RNA-seq reveals TOX as a key regulator of CD8^+^ T cell persistence in chronic infection. Nat Immunol.

[48] Dickinson BC, Chang CJ (2011). Chemistry and biology of reactive oxygen species in signaling or stress responses. Nat Chem Biol.

[49] Finkel T, Holbrook NJ (2000). Oxidants, oxidative stress and the biology of ageing. Nature.

[50] Wu H (2023). Mitochondrial dysfunction promotes the transition of precursor to terminally exhausted T cells through HIF-1α-mediated glycolytic reprogramming. Nat Commun.

[51] Peng HY (2021). Metabolic reprogramming and reactive oxygen species in T cell immunity. Front Immunol.

[52] Yu YR (2020). Disturbed mitochondrial dynamics in CD8^+^ TILs reinforce T cell exhaustion. Nat Immunol.

[53] Scharping NE (2016). The tumor microenvironment represses T cell mitochondrial biogenesis to drive intratumoral T cell metabolic insufficiency and dysfunction. Immunity.

[54] Bengsch B (2016). Bioenergetic insufficiencies due to metabolic alterations regulated by the inhibitory receptor PD-1 are an early driver of CD8(+) T cell exhaustion. Immunity.

[55] Abeynaike SA (2023). Human hematopoietic stem cell engrafted IL-15 transgenic NSG mice support robust NK cell responses and sustained HIV-1 infection. Viruses.

[56] Aryee KE (2022). Enhanced development of functional human NK cells in NOD-scid-IL2rg^null^ mice expressing human IL15. FASEB J.

[57] June CH (2018). CAR T cell immunotherapy for human cancer. Science.

[58] Deeks SG (2002). A phase II randomized study of HIV-specific T-cell gene therapy in subjects with undetectable plasma viremia on combination antiretroviral therapy. Mol Ther.

[59] Mitsuyasu RT (2000). Prolonged survival and tissue trafficking following adoptive transfer of CD4zeta gene-modified autologous CD4(+) and CD8(+) T cells in human immunodeficiency virus-infected subjects. Blood.

[60] Walker RE (2000). Long-term in vivo survival of receptor-modified syngeneic T cells in patients with human immunodeficiency virus infection. Blood.

[61] Beauparlant D (2017). Delineating CD4 dependency of HIV-1: adaptation to infect low level CD4 expressing target cells widens cellular tropism but severely impacts on envelope functionality. PLoS Pathog.

[62] Leibman RS (2017). Supraphysiologic control over HIV-1 replication mediated by CD8 T cells expressing a re-engineered CD4-based chimeric antigen receptor. PLoS Pathog.

[63] Anthony-Gonda K (2022). In vivo killing of primary HIV-infected cells by peripheral-injected early memory-enriched anti-HIV duoCAR T cells. JCI Insight.

[64] Anthony-Gonda K (2019). Multispecific anti-HIV duoCAR-T cells display broad in vitro antiviral activity and potent in vivo elimination of HIV-infected cells in a humanized mouse model. Sci Transl Med.

[65] Barber-Axthelm IM (2021). Stem cell-derived CAR T cells traffic to HIV reservoirs in macaques. JCI Insight.

[66] Carrillo MA (2017). New approaches for the enhancement of chimeric antigen receptors for the treatment of HIV. Transl Res.

[67] Zhen A (2017). Chimeric antigen receptor engineered stem cells: a novel HIV therapy. Immunotherapy.

[68] Zhen A (2016). Stem-cell based engineered immunity against HIV infection in the humanized mouse model. J Vis Exp.

[69] Mu W (2024). Examining chronic inflammation, immune metabolism, and T cell dysfunction in HIV infection. Viruses.

[70] Fenwick C (2019). T-cell exhaustion in HIV infection. Immunol Rev.

[71] Zhen A (2017). Targeting type I interferon-mediated activation restores immune function in chronic HIV infection. J Clin Invest.

[72] Mu W (2023). Targeting autophagy to treat HIV immune dysfunction. Autophagy Rep.

[73] Lee DJW (2024). Targeting ageing with rapamycin and its derivatives in humans: a systematic review. Lancet Healthy Longev.

[74] Rodes B (2022). Ageing with HIV: challenges and biomarkers. EBioMedicine.

[75] Babu H (2019). Systemic inflammation and the increased risk of inflamm-aging and age-associated diseases in people living with HIV on long term suppressive antiretroviral therapy. Front Immunol.

[76] Nasi M (2017). Ageing and inflammation in patients with HIV infection. Clin Exp Immunol.

[77] Henrich TJ (2024). Sirolimus reduces T cell cycling, immune checkpoint marker expression, and HIV-1 DNA in people with HIV. Cell Rep Med.

[78] Stock PG (2014). Reduction of HIV persistence following transplantation in HIV-infected kidney transplant recipients. Am J Transplant.

[79] Besnard E (2016). The mTOR complex controls HIV latency. Cell Host Microbe.

[80] Araki K (2009). mTOR regulates memory CD8 T-cell differentiation. Nature.

[81] Chi H (2012). Regulation and function of mTOR signalling in T cell fate decisions. Nat Rev Immunol.

[82] Varco-Merth BD (2022). Rapamycin limits CD4+ T cell proliferation in simian immunodeficiency virus-infected rhesus macaques on antiretroviral therapy. J Clin Invest.

[83] Kouro T (2022). Exhaustion of CAR T cells: potential causes and solutions. J Transl Med.

[84] Mu W (2022). Oral combinational antiretroviral treatment in HIV-1 infected humanized mice. J Vis Exp.

[85] Bolger AM (2014). Trimmomatic: a flexible trimmer for Illumina sequence data. Bioinformatics.

[86] Dobin A (2013). STAR: ultrafast universal RNA-seq aligner. Bioinformatics.

[87] Li H (2009). The Sequence Alignment/Map format and SAMtools. Bioinformatics.

[88] Subramanian A (2005). Gene set enrichment analysis: a knowledge-based approach for interpreting genome-wide expression profiles. Proc Natl Acad Sci U S A.

[89] Szklarczyk D (2019). STRING v11: protein-protein association networks with increased coverage, supporting functional discovery in genome-wide experimental datasets. Nucleic Acids Res.

