# Supplementary figures and images for: Rapamycin enhances CAR-T control of HIV replication and reservoir elimination in vivo
Source: J Clin Invest. 2025 Feb 11;135(7):e185489. doi: 10.1172/JCI185489 (PMC11957703; doi:10.1172/JCI185489)

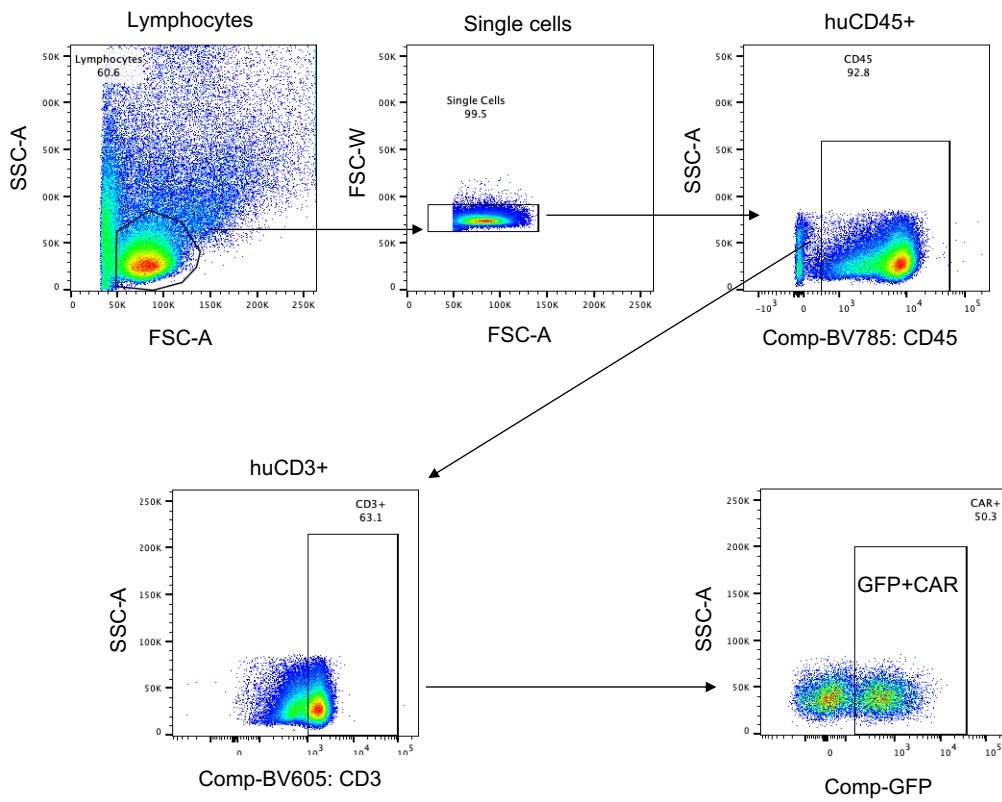

Supplement: Supplemental data [file jci-135-185489-s218.pdf]
